# *In vivo* pieces of the PP2A onco-puzzle fallen into place

**DOI:** 10.18632/oncoscience.384

**Published:** 2017-12-20

**Authors:** Bob Meeusen, Veerle Janssens

**Affiliations:** Laboratory of Protein Phosphorylation & Proteomics, Dept. of Cellular and Molecular Medicine, Faculty of Medicine, KU Leuven & LKI (Leuven Cancer Institute, KU Leuven), Leuven, Belgium

**Keywords:** B56 subunits, knockout mice, PP2A, PTPA, spontaneous tumorigenesis

Although kinases have hijacked the scene for several decades now in the battle against cancer, evidence has accumulated that phosphatases play equally important roles, by putting the brakes on hyperactivated oncogenic signaling associated with this disease. Since a long time, a major Ser/Thr-specific phosphatase, Protein Phosphatase 2A (PP2A), has been attributed important tumor suppressive properties, but clear *in vivo* evidence to sustain this notion was lacking. Recent work on the occurrence of spontaneous tumor formation in two independent PP2A-deficient mouse models finally filled this knowledge gap.

PP2A enzymes constitute a large family of >90 holoenzymes, each slightly or majorly structurally different, and hence, being differently regulated or harboring different functions [1]. Many PP2A complexes counteract signaling pathways driving growth, survival or protein synthesis, and are intrinsic regulators of diverse cell cycle checkpoints, contributing to their tumor suppressive roles in cells [1]. The nearly twenty-year- old suspicion that ‘PP2A’ represents an important tumor suppressor stems from its targeting by chemical tumor promotors, such as okadaic acid (OA), and by specific viral oncoproteins, such as SV40 and polyoma virus small t [1], which inhibit PP2A activity through a direct interaction with the catalytic C subunit (for OA), or with the scaffolding Aα subunit (for small t). In a subsequent effort to define the minimal genetic perturbations required for human epithelial cell transformation, it was found that expression of telomerase catalytic subunit (hTERT) and inhibition of tumor suppressors p53 and pRb (typically by SV40 large T) suffice for cell immortalization, while additional expression of an activated oncogene (e.g. H-RasV12) and inhibition of PP2A (typically by SV40 small t) are required to achieve full transformation [2]. It was further demonstrated that 50% suppression of PP2A Aα subunit or suppression of three PP2A regulatory B-type subunits (B56/B′α, B56/B′γ and B72/B”α1) could partially replace SV40 small t expression in cell transformation, while suppression of the PP2A C subunit or of the cellular PP2A activator PTPA had a much more substantial impact [3,4]. Hence, it was concluded that more than one tumor suppressive PP2A complex might need to be impaired to allow for full transformation.

A recent study by Sents et al. has now provided *in vivo* evidence to sustain the suspected importance of PP2A dysfunction in tumor initiation and/or progression [5]. In PTPA gene-trapped mice, still showing residual PTPA expression because of the hypomorphic nature of the PTPAgt allele, overall PP2A activity and C subunit methylation were reduced, while a selective decrease in activity of B56/B′ subunit-containing holoenzymes was revealed (B56/B′γ and B56/B′ε were tested) and activity of B55/B subunit-containing holoenzymes remained unaffected (only B55/Bα was tested). Interestingly, these mice exhibited higher rates of spontaneous tumor formation than wild-type mice. The observed neoplasms were mainly hematologic malignancies and sporadically, hepatocellular adeno(carcino)mas. Although not identical throughout tumor samples, probably due to the random nature of the oncogenic event, activation of diverse oncogenic pathways was observed, including an increase in c-Myc phosphorylation, and in expression of β-catenin (Wnt signaling) and Gli-1 (Hedgehog signaling), all oncogenes known from cellular studies to be suppressed by B56/B′-type of PP2A holoenzymes. Importantly, analysis of cBioportal and COSMIC cancer databases demonstrated a high frequency (up to 70%) of heterozygous loss or monoallelic loss-of-function mutation of the PTPA- encoding gene *PPP2R4* in a strikingly large set of cancers. In some cases, this correlated with significantly decreased overall patient survival, further underscoring the clinical importance of *PPP2R4* heterozygous loss or mutation as a novel high-penetrance genetic mechanism of PP2A inactivation. Together, these data established *PPP2R4* as a novel obligate haploinsufficient tumor suppressor gene [5].

Although the former study seems to confirm that likely more than one PP2A holoenzyme needs to be functionally disturbed to achieve abolishment of the tumor suppressor function of PP2A, another recent study by Lambrecht et al. convincingly demonstrated that even the lack of a single PP2A regulatory B56/B′-type subunit can promote spontaneous tumorigenesis *in vivo* [6]. Indeed, *Ppp2r5d* knockout mice, devoid of the PP2A B56/B′δ subunit in all tissues, appeared prone to spontaneous development of diverse cancer types, including hematologic malignancies, and much more surprisingly, hepatocellular carcinomas (HCC). In the oldest age group of the knockout mice, HCC incidence reached nearly 60% and could not be linked to increased liver inflammation or steatosis, as nearly 70% of the tumors arose in a normal liver context. All HCCs examined showed increased c-Myc Ser62 phosphorylation, as well as GSK-3β Ser9 phosphorylation, the latter being also found in the healthy knockout livers. Thus, it was proposed that loss of PP2A-B56/B′δ-mediated dephosphorylation of GSK- 3β predisposed for increased c-Myc oncogenicity upon fortuitous Ser62 phosphorylation by preventing c-Myc degradation. Surprisingly, no obvious clinical evidence was found for direct genetic inactivation of *PPP2R5D* in human HCCs or other cancer types, feeding the authors' hypothesis that impairment of PP2A-B56/B′δ function may largely occur through indirect mechanisms in human cancers, e.g. through specific mutations in *PPP2R1A*, encoding the Aα subunit [7].

In conclusion, two recent mouse genetics studies [5,6] eventually provided firm *in vivo* evidence to sustain the tumor suppressive role of PP2A-B56/B′ holoenzymes, and particularly of the PP2A-B56/B′δ complex. This paves the way for the full clinical development of the PP2A status in tumors as prognostic or predictive markers, and for the exploitation of dysfunctional PP2A as a promising new target for reactivation therapies [8].

**Figure 1:**
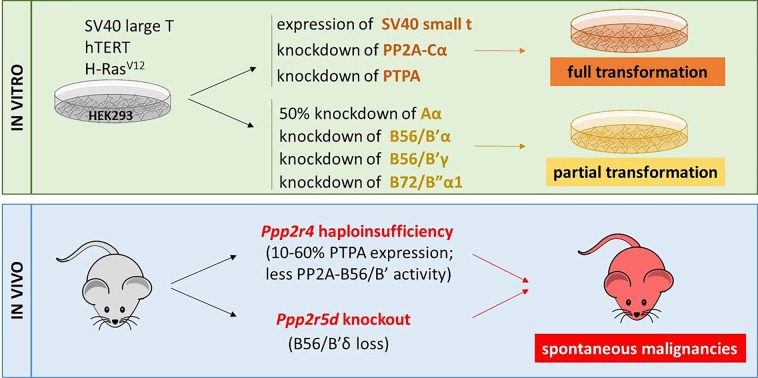
Downregulation of specific PP2A subunits or PP2A regulators results in human epithelial cell transformation *in vitro*, or spontaneous tumorigenesis *in vivo* – highlighting the major importance of PP2A dysfunction in tumor initiation and/or progression.

## References

[1] Janssens (2005). Curr Opin Genet Dev.

[2] Hahn WC (2002). N Engl J Med.

[3] Chen W (2005). Cancer Res.

[4] Sablina A (2010). Cancer Res.

[5] Sents W (2017). Cancer Res.

[6] Lambrecht (2017). Oncogene.

[7] Haesen D (2016). Cancer Res.

[8] Meeusen B (2017). Int J Biochem Cell Biol.

